# Supplementary figures and images for: Substantial acetylcholine reduction in multiple brain regions of Mecp2-deficient female rats and associated behavioral abnormalities
Source: PLoS One. 2021 Oct 21;16(10):e0258830. doi: 10.1371/journal.pone.0258830 (PMC8530288; doi:10.1371/journal.pone.0258830)

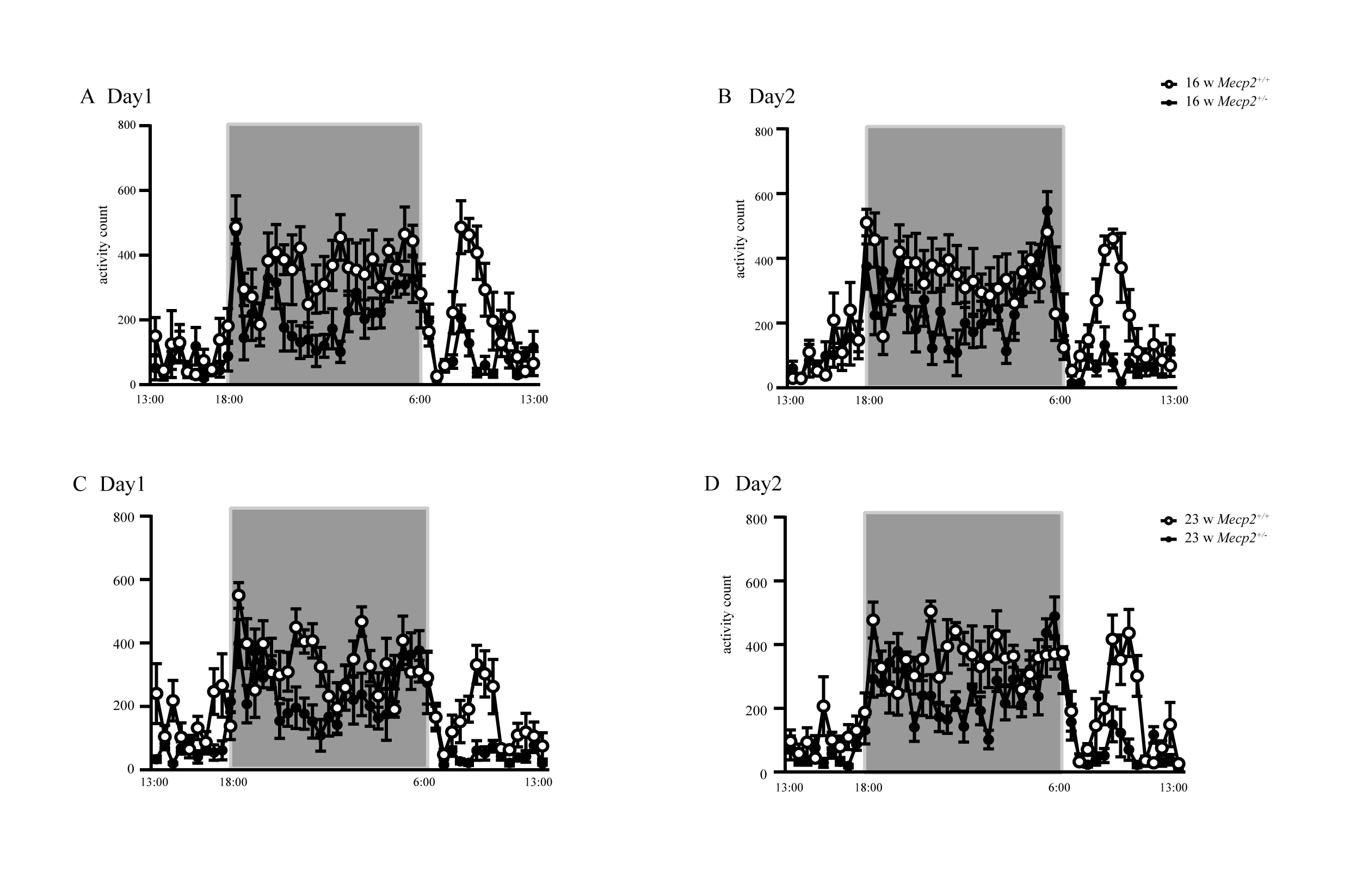

Supplement: S1 Fig — A-D: 24-h locomotor activity in female Mecp2+/+ (open symbols) and Mecp2+/- (filled symbols) rats. Data are the average ± SE of data collected at each half an hour from 16 w (A, B) or 23 w (C, D) old rats over 2 consecutive 24-h periods (A, C: day1; B, D: day2) (n = 6, for each geneotype). Non significance female Mecp2+/- vs. Mecp2+/+ rats (Kruskal–Wallis with Dunn’s post hoc multiple comparisons test). (TIF) [file pone.0258830.s001.tif]
