# Supplementary material for: Substantial acetylcholine reduction in multiple brain regions of Mecp2-deficient female rats and associated behavioral abnormalities
Source: PLoS One. 2021 Oct 21;16(10):e0258830. doi: 10.1371/journal.pone.0258830 (PMC8530288; doi:10.1371/journal.pone.0258830)
Supplement: Attachment — Submitted filename: draft review 210728 2204 2147 PONE-D-21-05550R1.pdf [file pone.0258830.s003.pdf]

Draft Review
July 28, 2021
Substantial acetylcholine reduction in multiple brain regions of *Mecp2*-deficient female rats and associated behavioral abnormalities
PONE-D-21-05550R1

The authors compared and contrasted the neurochemical and behavioral aspects of wild type and *Mecp2*-deficient female rats utilizing a reasonable experimental design and analysis. They concluded that acetylcholine concentration was associated with behavior.

The study was well conducted. The discussion was reasonable.

Some readers may be unfamiliar with some technical details so explicitly documenting them will likely facilitate understanding. Some sections may be better worded and organized to help readers comprehend the key points.

The manuscript will likely merit publication with revisions.

Page 6
Line 131
Change “MWM test” to “Morris water maze (MWM) test”

Page 7
Line 157
Change “Friedman’s test” to “Friedman test”
Provide a reference for the Friedman test. What statistical program was used?
Line 161
Provide a reference for unpaired two-tailed Mann–Whitney U-test. What statistical program was used?

Page 11
Lines 245-246
Provide a reference for unpaired two-tailed Mann–Whitney U-test.
Line 247
Provide a reference for the Kruskal–Wallis with Dunn’s *post hoc* multiple comparisons test.
Line 248
Provide a reference for the Friedman test.

Page 12
Line 269
Provide a reference for the Friedman test.
Line 270
Provide a reference for the Kruskal–Wallis with Dunn’s *post hoc* multiple comparisons test.
Line 277
Provide a reference for the Kruskal–Wallis with Dunn’s *post hoc* multiple comparisons test.

Page 13
Lines 292-293
Provide a reference for the Kruskal–Wallis with Dunn’s *post hoc* multiple comparisons test.
Lines 298-299
Provide a reference for unpaired two-tailed Mann–Whitney U-test.
Line 308
Change “Fig-2D” to “Fig 2D”

Page 15
Line 327
Change “Mecp2-deficient.” to “Mecp2-deficient”

Page 16
None of the items utilize **.
Line 331
Change “*p < 0.05 and **p < 0.01 vs. female Mecp2+/+” to
“*p < 0.05 female *Mecp*$^{2+/-}$ vs*.* *Mecp*$^{2+/+}$”
Lines 331-332
Provide a reference for unpaired two-tailed Mann–Whitney U-test.

Page 17
Table 2
None of the items utilize *.
Line 335
Change *p < 0.05 vs. female Mecp2+/+ rats” to
*Non significance female *Mecp*$^{2+/-}$ vs. *Mecp*$^{2+/+}$ rats”
Lines 335-336
Provide a reference for unpaired two-tailed Mann–Whitney U-test.
Line 339
Change “vs. female Mecp2+/+ rats” to “female *Mecp*$^{2+/-}$ vs. *Mecp*$^{2+/+}$ rats”
Provide a reference for unpaired two-tailed Mann–Whitney U-test.

Page 19
Line 361
Change **p <0.01 vs. female Mecp2+/+ rats” to **p <0.01 female *Mecp*$^{2+/-}$ vs. *Mecp*$^{2+/+}$ rats”
Line 362
Provide a reference for unpaired two-tailed Mann–Whitney U-test.
Line 366
Change “**p < 0.01 vs. female Mecp2+/+ rats” to “**p < 0.01 female *Mecp*$^{2+/-}$ vs. *Mecp*$^{2+/+}$ rats”

Page 20
Line 385
Change “demonstrate” to “demonstrated”

Abbreviations
Place the abbreviations in alphabetical order.
