# Supplementary material for: Substantial acetylcholine reduction in multiple brain regions of Mecp2-deficient female rats and associated behavioral abnormalities
Source: PLoS One. 2021 Oct 21;16(10):e0258830. doi: 10.1371/journal.pone.0258830 (PMC8530288; doi:10.1371/journal.pone.0258830)
Supplement: Attachment — Submitted filename: Response to Reviewer_210602.docx [file pone.0258830.s002.docx]

**Response to Reviewers:**

We wish to express our appreciation to the Reviewers for their insightful comments, which have helped us improve our paper substantially.

**Reply to Reviewer #1**

**Comments to authors**

**Point 1: Materials and methods Morris water maze test Line 127: Since the second figure to be mentioned in the text occurs in this line, the figure should be numbered figure 2.**

Answer 1: Thank you for the correction. We have accordingly altered the order of the figure number.

**Point 2: Materials and methods Statistical Analysis: Are the data normally distributed as demonstrated by the Kolmogorov-Smirnov or Wilks-Shapiro procedures? If the data are not normally distributed, then a nonparametric procedure such as Kruskal-Wallis is appropriate.**

Answer 2: Thank you for the comment. Because sample size for each experiment was small, it was not appropriate to evaluate the equal distribution of the sample. We therefore changed all our statistical methods to nonparametric procedures, accordingly.

**Point 3:** **Results Impaired social behaviors in Mecp2-deficient female rats: Page 9 Line 192 Change “disorders” to “disorder”.**

Answer 3: Thank you for the correction. We have accordingly changed “disorders” to “disorder.”

**Point 4: Table 1 Pages 13 to 15 Express the values of each row in a single line by using a smaller font.**

Answer 4: Thank you for the correction. In accordance, we have corrected the table to express the values of each row in a single line using a smaller font.

**Point 5: Legend to figure 7 Line 316 The scale bar appears to be the same length in all panels. What is the magnification indicated by the two scale bars?**

Answer 5: Thank you for the correction. In accordance, we have corrected the scale bars.

**Point 6: Discussion Relationships between cholinergic and behavioral abnormalities Line 387-408 Include a discussion of the dopaminergic deficits observed as follows: Wong DF, Blue ME, Brašić JR,, Nandi A, Valentine H, Stansfield KH, Rousset O, Bibat G, Yablonski ME, Johnston MV, Gjedde A, Naidu SB. Are dopamine receptor and transporter changes in Rett syndrome reflected in Mecp2-deficient mice? Exp Neurol. 2018; 307: 74-81. https://doi.org/10.1016/ j.expneurol.2018.05.019 PubMed PMID: 29782864.**

Answer 6: Thank you for the comment and information. In accordance, we have thoroughly corrected the part of the Discussion section concerning the possible involvement of dopaminergic deficits in the behavioral abnormalities of Mecp2^+/−^ rats and cited the article.

**Point 7:** **Reference Line 517 Remove the space between “30.” and “Panayotis”**

Answer 7: Thank you for the correction. We have accordingly removed the space between “30.” and “Panayotis.”

**Point 8:** **Lines 549-550 Reference 41. Include the original title of the article in German as follows: Rett A. Uber ein bisher nicht bekanntes Krankheitsbild einer angeborenen Stoffwechselst örung [On an until now unknown disease of a congenital metabolic disorder]. Krankenschwester. 1966 Sep;19(9):121-2. German. PMID: 5179620.**

Answer 8: Thank you for the correction. In accordance, we have corrected the reference to include the original title of the article in German.

**Point 9:** **Please add appendix of abbreviations. Define all acronyms before their first use.**

Answer 9: Thank you for the correction. In accordance, we have added an appendix of abbreviations and defined all acronyms before their first use.

**Point 10:** **Supplemental Methods The section in the supplemental methods belong in the body of the text. There are no restrictions on word counts, number of figures, or amount of supporting information.**

Answer 10: Thank you for the comment. In accordance, we have moved the supplemental methods into the body of the text.

**Reply to reviewer #2**

**Comments to authors**

**Point 1: The correlation of behavioral changes with acetylcholine levels is appears far reaching based on the way the manuscript is organized.**

Answer 1: Thank you for the comment. Considering both reviewers’ comments, we have thoroughly corrected the part of the Discussion section concerning the possible involvement of monoaminergic dysfunctions in the behavioral abnormalities of the Mecp2^+/−^ female rats.

**Point 2: Figure 7 images are of poor quality and images of amygdala and hypothalamus should be included.**

Answer 2: Thank you for the comment. In accordance, we have replaced the microscopic photographs and also inserted images of the amygdala and hypothalamus in Figure 7 and Figure 8.

**Point 3: Page 2, Line 25. “…..spatial cognition as well lower plasma” - the word “as” is missing.**

Answer 3: Thank you for the correction. In accordance, we have added the word “as.”

**Point 4: Page 9, Line 193. The author report a decrease in repetitive grooming behavior. Veeraragavan et al. 2016 reported no significant changes in Mecp2-/+ mice, what do you attribute to the divergent results?**

Answer 4: Thank you for the comments. Concerning self-grooming, we considered that the divergent results would come from the developmental impairment in the cholinergic system. Excessive self-grooming behavior, however, is a typical symptom of autism model mice and has also been observed in Mecp2 null male mice mutants. Therefore, further investigations are necessary to understand the meaning of this behavioral abnormality in the Mecp2-deficient female rat. We have added these limitations to the Discussion section.

**Point 5: Page 22, Line 403: “….GABAA (GABAnergic….” – should be** **“GABAergic”**

Answer 5: Thank you for the correction. In accordance, we have corrected the word to “GABAergic.”

**Point 6: Figure 4 A and B. For consistency capitalize the first word on the y axis.**

Answer 6: Thank you for the correction. In accordance, we have capitalized the first word on the y-axis.

**Point 7: Figure 6 B and C. For consistency capitalize the words on the y axis “goal latency” and “swimming distance”.**

Answer 7: Thank you for the correction. In accordance, we have capitalized the first word on the y-axis.
