# Supplementary material for: Substantial acetylcholine reduction in multiple brain regions of Mecp2-deficient female rats and associated behavioral abnormalities
Source: PLoS One. 2021 Oct 21;16(10):e0258830. doi: 10.1371/journal.pone.0258830 (PMC8530288; doi:10.1371/journal.pone.0258830)
Supplement: Attachment — Submitted filename: draft review 210728 2204 2147 PONE-D-21-05550R1.docx [file pone.0258830.s004.docx]

Draft Review

July 28, 2021

Substantial acetylcholine reduction in multiple brain regions of *Mecp2*-deficient female rats and associated behavioral abnormalities

PONE-D-21-05550R1

The authors compared and contrasted the neurochemical and behavioral aspects of wild type and *Mecp2*-deficient female rats utilizing a reasonable experimental design and analysis. They concluded that acetylcholine concentration was associated with behavior.

The study was well conducted. The discussion was reasonable.

Some readers may be unfamiliar with some technical details so explicitly documenting them will likely facilitate understanding. Some sections may be better worded and organized to help readers comprehend the key points.

The manuscript will likely merit publication with revisions.

Page 6

Line 131

Change “MWM test” to “Morris water maze (MWM) test”

Page 7

Line 157

Change “Friedman’s test” to “Friedman test”

Provide a reference for the Friedman test. What statistical program was used?

Line 161

Provide a reference for unpaired two-tailed Mann–Whitney U-test. What statistical program was used?

Page 11

Lines 245-246

Provide a reference for unpaired two-tailed Mann–Whitney U-test.

Line 247

Provide a reference for the Kruskal–Wallis with Dunn’s *post hoc* multiple comparisons test.

Line 248

Provide a reference for the Friedman test.

Page 12

Line 269

Provide a reference for the Friedman test.

Line 270

Provide a reference for the Kruskal–Wallis with Dunn’s *post hoc* multiple comparisons test.

Line 277

Provide a reference for the Kruskal–Wallis with Dunn’s *post hoc* multiple comparisons test.

Page 13

Lines 292-293

Provide a reference for the Kruskal–Wallis with Dunn’s *post hoc* multiple comparisons test.

Lines 298-299

Provide a reference for unpaired two-tailed Mann–Whitney U-test.

Line 308

Change “Fig-2D” to “Fig 2D”

Page 15

Line 327

Change “Mecp2-deficient.” to “Mecp2-deficient”

Page 16

None of the items utilize **.

Line 331

Change “*p < 0.05 and **p < 0.01 vs. female Mecp2+/+” to

“*p < 0.05 female *Mecp^2+/-^* vs*. Mecp^2+/+^*”

Lines 331-332

Provide a reference for unpaired two-tailed Mann–Whitney U-test.

Page 17

Table 2

None of the items utilize *.

Line 335

Change *p < 0.05 vs. female Mecp2+/+ rats” to

*Non significance female *Mecp^2+/-^* vs. *Mecp^2+/+^* rats”

Lines 335-336

Provide a reference for unpaired two-tailed Mann–Whitney U-test.

Line 339

Change “vs. female Mecp2+/+ rats” to “female *Mecp^2+/-^* vs. *Mecp^2+/+^* rats”

Provide a reference for unpaired two-tailed Mann–Whitney U-test.

Page 19

Line 361

Change **p <0.01 vs. female Mecp2+/+ rats” to **p <0.01 female *Mecp^2+/-^* vs. *Mecp^2+/+^* rats”

Line 362

Provide a reference for unpaired two-tailed Mann–Whitney U-test.

Line 366

Change “**p < 0.01 vs. female Mecp2+/+ rats” to “**p < 0.01 female *Mecp^2+/-^* vs. *Mecp^2+/+^* rats”

Page 20

Line 385

Change “demonstrate” to “demonstrated”

Abbreviations

Place the abbreviations in alphabetical order.
