# Supplementary material for: Substantial acetylcholine reduction in multiple brain regions of Mecp2-deficient female rats and associated behavioral abnormalities
Source: PLoS One. 2021 Oct 21;16(10):e0258830. doi: 10.1371/journal.pone.0258830 (PMC8530288; doi:10.1371/journal.pone.0258830)
Supplement: Attachment — Submitted filename: Response to Reviewer_210830.docx [file pone.0258830.s005.docx]

**Response to Reviewers:**

We wish to express our appreciation to the Reviewers for their insightful comments, which have helped us improve our paper substantially.

**Reply to Reviewer #1**

**Point 1: Page 6**

**Line 131 Change “MWM test” to “Morris water maze (MWM) test”.**

Answer 1: Thank you for the correction. We have accordingly spelled out “MWM test”.

**Point 2: Page 7**

**Line 157 Change “****Friedman’s test” to “Friedman test” Provide a reference for the Friedman test. What statistical program was used?**

**Line 161 Provide a reference for unpaired two-tailed Mann–Whitney U-test. What statistical program was used?**

Answer 2: Thank you for the comment. We have accordingly changed “Friedman’s test” to “Friedman test” Concerning about statistical program, we had used GaphPad Prism (version 9.1.2 for Windows) for all statistical analyses (Friedman test, unpaired two-tailed Mann-Whitney U-test, and Kruskal-Wallis with Dunn’s *post hoc* multiple comparisons test), according to the software guide. So, we have described it in the revised manuscript.

**Point 3:** **Page 13**

**Line 308 Change “Fig-2D” to “Fig 2D”**

Answer 3: Thank you for the correction. We have accordingly changed “Fig-2D” to “Fig 2D”.

**Point 4: Page 15**

**Line 327 Change** **“Mecp2-deficient.” to “Mecp2-deficient**

Answer 4: Thank you for the correction. We have accordingly changed “Mecp2-deficient.” to “Mecp2-deficient”.

**Point 5:** **Page 16**

**Table 1 None of the items utilize **.**

**Line 331 Change “*p < 0.05 and **p < 0.01 vs. female Mecp2^+/+^” to “*p < 0.05 female Mecp2^+/-^ vs. Mecp2*^+/+^*”**

Answer 5: Thank you for the correction. In accordance, we have changed “*p < 0.05 and **p < 0.01 vs. female Mecp2^+/+^” to “*p < 0.05 female Mecp2^+/-^ vs. Mecp2^+/+^”.

**Point 6: Page 17**

**Table 2 None of the items utilize *.**

**Line 335** **Change *p < 0.05 vs. female Mecp2^+/+^ rats” to *Non significance female Mecp2^+/-^ vs. Mecp2^+/+^ rats”.**

**Line 339 Change** **“vs. female Mecp2^+/+^ rats” to “female Mecp2^+/-^ vs. Mecp2^+/+^ rats”**

Answer 6: Thank you for the correction. In accordance, we have changed *p < 0.05 vs. female Mecp2^+/+^ rats” to *Non significance female Mecp2^+/-^ vs. Mecp2^+/+^ rats”. We also have changed “vs. female Mecp2^+/+^ rats” to “female Mecp2^+/-^ vs. Mecp2^+/+^ rats”.

**Point 7:** **Page 19**

**Line 361** **Change **p <0.01 vs. female Mecp2^+/+^ rats” to **p <0.01 female Mecp2^+/-^ vs. Mecp2^+/+^ rats”**

**Line 366 Change “**p < 0.01 vs. female Mecp2+/+ rats” to “**p < 0.01 female Mecp2+/- vs. Mecp2+/+ rats”**

Answer 7: Thank you for the correction. In accordance, we have changed **p <0.01 vs. female Mecp2^+/+^ rats” to **p <0.01 female Mecp2^+/-^ vs. Mecp2^+/+^ rats”. We also have changed “**p < 0.01 vs. female Mecp2^+/+^ rats” to “**p < 0.01 female Mecp2^+/-^ vs. Mecp2^+/+^ rats”.

**Point 8:** **Page 20**

**Line 385 Change “****demonstrate” to “demonstrated”.**

Answer 8: Thank you for the correction. We have accordingly changed “demonstrate” to “demonstrated”.

**Point 9:** **Abbreviations Place the abbreviations in alphabetical order.**

Answer 9: Thank you for the correction. In accordance, we have replaced the abbreviations in alphabetical order.

**Reply to reviewer #2**

**Comments to authors**

**Point 1: Page 4, Line 78. Remove** **“on so on”.**

Answer 1: Thank you for the correction. In accordance, we have removed “on so on”.

**Point 2: Page 7, Line 150. This is the Figure legend for figure 2, not sure why it is in the methods section.**

Answer 2: Thank you for the comment. Since we had mentioned the figure in the materials and methods section “Morris water maze (MWM) test”, the reviewer#1 had advised the figure to be numbered figure 2 in the previous comments, according to the Plos One’s instructions. However, since the mismatch between the order of figures and results could confuse readers, we have replaced the Fig 2 after the result section “Impaired spatial learning and memory in Mecp2-deficient female rats” and altered the order of the figure number.

**Point 3:** **Page 12, Line 260-261. “Both the frequency (Fig 4A) and duration 261 (Fig. 4B) of self-grooming behavior were significantly or tended to be reduced in female Mecp2^+/−^…” …… The current phrasing is not clear, authors should clarify that frequency was reduced and duration tended to be reduced but not significant.**

Answer 3: Thank you for the correction. In accordance, we have revised the sentence to clarify that frequency was reduced and duration tended to be reduced but not significant.

**Point 4:** **Page 12, Line 283. “Spontaneous locomotor activity was significantly or tended to be lower among female Mecp2^+/−^ rats compared to female Mecp2^+/+^ rats (Fig 5) during both light and dark phases.” Authors should clarify the specific timepoints of significance.**

Answer 4: Thank you for the comments. We have shown the locomotor activities at each 30-min from 16 w or 23 w old rats over 2 consecutive 24-periods as supplemental S1 Fig in the revised manuscript. After statistical analyses (Kruskal–Wallis with Dunn’s *post hoc* multiple comparisons test), we have found no significant difference between genotypes at any time point. These results indicated that the *Mecp2* deficiency is not likely to affect circadian rhythms but locomotor activity itself. So, we have additionally described it in the revised manuscript.

**Point 5: Page 12, Line 285. For consistency lower case w “29 w”**

Answer 5: Thank you for the correction. We have accordingly changed “29W” to “29 w”.

**Point 6: Page 13, Line 301. It is unclear why** **the result section “Impaired spatial learning and memory in Mecp2-deficient female rats” is Figure 2, following Figure 6.**

Answer 6: Thank you for the correction. In accordance, we have revised this matter as described in Point 2.

**Point 7: Page 14**

**Line 314. “Surprising, however, biogenic amine and…” – Change to “Surprisingly”**

**Line 323. “These results suggest that impaired cholinergic signaling would severely affect the behavioral abnormalities observed...” The word “would” should be changed to “could”.**

Answer 7: Thank you for the correction. In accordance, we have changed “Surprising” to “Surprisingly”, and “would” to “could”.

**Point 8: Page 18, Line 344.” Astrocytes are known to support neuronal function....” A reference is needed for this sentence.**

Answer 7: Thank you for the correction. In accordance, we have added references for the sentence.

**Point 9: Page 18, Line 355. “Both the neurotrophic factors play important…” – Remove “the”.**

Answer 9: Thank you for the correction. In accordance, we have removed “the”.
